# Anesthetic and airway management of a child with a large upper-lip hemangioma

**DOI:** 10.4103/1658-354X.76479

**Published:** 2011

**Authors:** Sukhminderjit Singh Bajwa, Aparajita Panda, Sukhwinder Kaur Bajwa, Amarjit Singh, S. S. Parmar, Kanwalpreet Singh

**Affiliations:** *Department of Anaesthesiology and Intensive Care, Gian Sagar Medical College & Hospital, Ram Nagar, Banur, Punjab, India*; 1 *Department of Obstetrics and Gynaecology, Gian Sagar Medical College & Hospital, Ram Nagar, Banur, Punjab, India*

**Keywords:** *Airway management*, *difficult airway*, *hemangioma*, *hemangioma upper lip*, *intralesional steroids*

## Abstract

An 11-month-old male child weighing 8 kg was brought to the plastic surgery out-patient department by his parents with chief complaints of sudden increase in size of a swelling over the upper lip and difficulty in feeding for the last 7 days. It was diagnosed as a case of hemangioma of the upper lip. All the routine and special investigations including coagulation profile of the child were normal. The child was planned for ablation of feeding vessels along with intralesional steroid injection. Airway management of the child posed the challenge for us as the size and site of the lesion carried the risk of difficult intubation and possible risk of extensive hemorrhage. All the requisite equipment for difficult airway management was made ready. We were able to intubate the child with miller number-2 blade from the left angle of mouth without putting much pressure on the swelling. The surgical and postoperative period was uneventful and the child was discharged the next day to be followed up after 2 weeks.

## INTRODUCTION

The hemangiomas of the face, head and neck region represents almost one-third of all hemangiomas in humans. The incidence in newborn is 1-3% and it progressively increases with age up to 1 year when its incidence increases to 10%.[1] Among them, oral lesions are very common and represent about 14% of all human hemangiomas.[2] The anesthetic consideration mainly stresses upon the blood loss. But if the site of lesion involves the facial or oral tissues, then airway management acquires a prime importance along with the management of possible hemorrhagic loss. We are reporting a case of a large hemangioma of upper lip in a child of 11 months who was brought to our institute by his parents for the surgical treatment of the lesion.

## CASE REPORT

**Figure 1:**
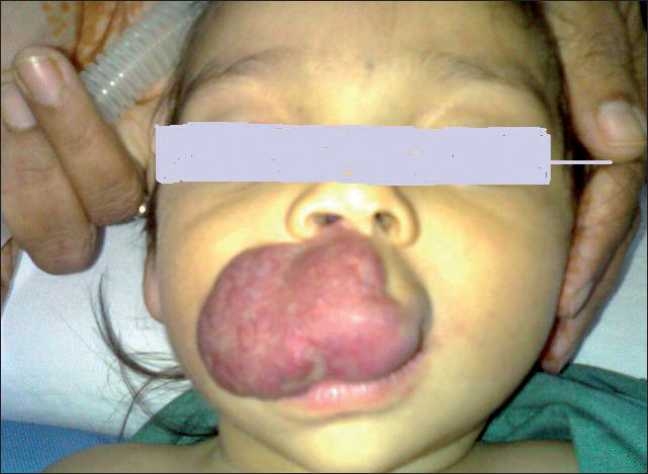
Frontal view picture of the child showing the large sized haemangioma of the upper lip

**Figure 2:**
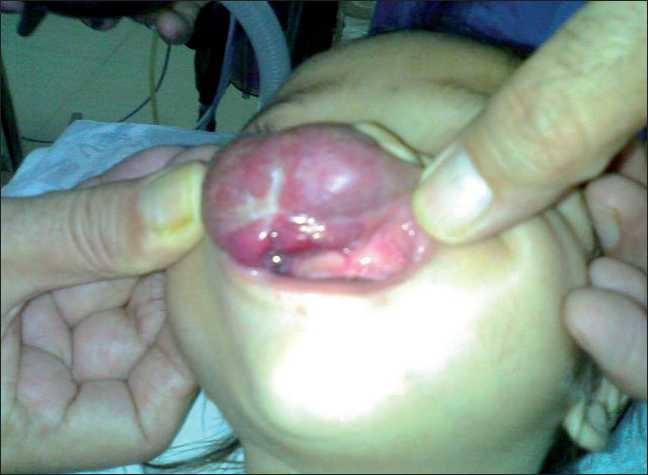
Anterio-inferior view of the face showing difficult mouth opening due to the large haemangioma of upper lip

An 11-month-old male child was brought to the plastic surgery OPD of our institute by his parents with the chief complaints of increase in size of a swelling over upper lip with difficulty in oral intake for the last 1 week. On eliciting the history, it was revealed that the lesion was present since birth and had been gradually enlarging. The size of the lesion was measured to be 5.5×3.5×1.5 cm and was diagnosed as a case of hemangioma upper lip [Figures 1 and 2]. Previously, the patient did not have much difficulty in feeding but the difficulty rose since 1 week as the lesion had suddenly enlarged. He was being fed by the spoon from the angle of the mouth. Considering the age of the child, size of the lesion and most importantly the site of the hemangioma, it was planned to ablate and strangulate the vessels during the first stage so as to decrease the size of lesion before proceeding for surgical excision. Preanesthetic assessment revealed a very narrow mouth opening with difficulty in assessing the mallampatti grading [Figures 1 and 2]. All the investigations were within normal limits including the coagulogarm and weight of the child was found to be 8 kg.

General anesthesia was planned for the child with main stress upon airway management. The child was kept in a fasting state for 6 hours and was administered 2 mg of syrup midazolam 30 minutes before the surgical procedure. Airway trolley was made ready with all the pediatric airway equipment including the fibreoptic bronchoscope of 2.4 mm diameter. The child was taken to the operation theatre by one female staff who had been with him for the last half-an-hour and had developed a good rapport with the child. Pulse oximetry probe was attached to the great toe of right foot and a pediatric stethoscope was fixed on the precordium. Induction of anesthesia was achieved with sevoflurane in 6 L of oxygen administered through number 3 face mask attached to Jackson Rees circuit while covering the eyes with soft cotton pad to prevent injury to the eyes. A good intravenous access was secured with 22 G venflow and after establishing a good breathing pattern evident from the bag movements we administered 15 mg of ketamine. When we were sure that we can easily ventilate the patient, we administered 20 mg of succinylcholine while sevoflurane was kept at 2.5% during this stage. Gentle positive pressure ventilation was carried out for 1 minute. Laryngoscopy was done with miller blade size 2 from the left corner of the mouth and tongue was lifted in a diagonal manner and we were able to see the full view of glottis. During the whole exercise of laryngoscopy the fulcrum on the upper teeth was never made rather a forward and upward pressure was maintained without even touching the mass. An oral RAE endotracheal tube number 4.5 mm (internal diameter) was inserted from the right corner of the mouths while an assistant helped continuously in raising the mass with a soft cotton pad with gentle force so as to enable laryngoscopy and a good intraoral view. After checking the equal air entry bilaterally, we packed the oral cavity with a roll gauge to prevent any aspiration of secretions or possibly blood as the tube was without a protective cuff. The child was then paralyzed with 3 mg of injection atracurium and positive pressure ventilation was carried out throughout the procedure with Jackson Rees circuit with nitrous oxide and oxygen in the ratio of 60:40. Right from the beginning a team of plastic surgeon, a general surgeon and an otorhinolaryngeologist were scrubbed and ready with their equipment. Surgical procedure was initiated and peripheral surgical ablation of the feeding vessels was done with electric cauterization. Thereafter the intralesional injection of steroid was administered. The surgical procedure lasted for 15 minutes and at the end of surgery the child was reversed with 0.5 mg of injection neostigmine and 0.1 mg of glycopyrrolate. The dry intraoral roll gauge pack was removed and the child was extubated in a fully awake state after establishment of adequate and rhythmic respiratory efforts with good tidal volume and flexion of legs at the hip joint. He was given rectal suppository of 150 mg paracetamol for postoperative pain relief after induction of anesthesia. The child was kept in the recovery ward for 2 hours and later on shifted to the pediatric wing of the plastic surgery ward where he was kept for another 1 day and was discharged after an uneventful recovery period during the hospital stay. The parents were instructed for follow-up of the child after 15 days.

## DISCUSSION

Hemangiomas are usually present at birth but may develop later on also but most of them arise because of developmental anomalies.[3] The mucosal hemangioma is characterized by a well-circumscribed, soft, painless lesion which is blue or red in appearance. The growth of these lesions in the elderly is quite slow but congenital lesions keep up a good pace with the physical growth. Sometimes, the overlying lesion can cause compression symptoms on the underlying tissues causing pressure atrophy of the tissues and bones. Pathologically, it is characterized by abundance of intertwined capillaries or veins which helps in their structural classification either into capillary or cavernous hemangioma. The anastomosis between capillaries and veins can lead to formation of arteriovenous hemangioma. Usually, these hemangiomas are asymptomatic, but their presence especially on facial region has got numerous cosmetic implications and considerations.[4] It brings a feeling of inferiority complex in the grown-ups as it causes aesthetic deformities while in children it can be the reason for teasing among the peer group. If the lesion is fast growing and involves the mucosa of lip, it can interfere with the feeding or can block the opening of nostrils thus causing difficulty in breathing.[5] Management of such pediatric patients, who present for surgery, involves a lot of anesthetic and surgical considerations.

The oral lesions, especially of lips, provide a very difficult situation for the attending anesthesiologist. The airway management of such challenging cases has to be planned precisely especially in the pediatric age group. The limited number of airway gadgets and equipment to deal with difficult airway in pediatric population, difficulty in managing the anatomical and functional aspects of pediatric airway and the decreased cardiorespiratory reserve makes the job of an anesthesiologist hugely challenging. To compound the matters further, if some lesion is present on any part of this anatomical track the degree of difficulty increases manifold.[5] In our case, this degree of difficulty was associated with many other challenges also. Not only the site of lesion was a major problem but its size too added much scores to the predicted difficulty. All the more, it was a highly vascular lesion and any pressure or fiddling with it during the airway management could have caused uncontrolled bleeding from it. That’s why we were ready with the pediatric fibreoptic bronchoscope but the only problem with it was that the sliding of tube number 4.5 was quite difficult. Laryngeal mask airway was another option but it was also fraught with dangers of dislodgement and risk of aspiration during the surgical procedure. We decided to proceed with laryngoscopy as we were encouraged by a good breathing pattern during the initial stages of induction of anesthesia. We choose miller straight blade rather than curved one as the latter was difficult to use from the left corner of the mouth.

From surgical point of view, various options include intralesional injection of sclerosing agents or steroids, laser ablation, cryosurgery, implantation or external irradiation and ablation of feeding vessels.[6]

The age of the patient, size of the lesion, site of the hemangioma, the clinical back-up facilities like presence of vascular surgeon and the intensive care as well as cosmetic considerations determines the type of surgical intervention to be undertaken. In this case peripheral surgical cauterization was done followed by intralesional injection of triamcinolone acetonide and betamethasone acetate. The peripheral electric cauterization was aimed at ablation of feeding vessels while steroids have a huge success rate, to the extent of 77% in some cases in the involution of hemangiomas.[7–9] A good cooperation between the surgical and the anesthesia team is required for the successful treatment of such giant lesions. An insightful surgeon and an experienced and skilled anesthetist are the prerequisites of such surgical procedures especially in the pediatric age group. The protocols and guidelines for difficult airway management in pediatric age groups have to be followed while dealing with such challenging cases so as to prevent any catastrophes as pediatric patients give a very little time for resuscitation due to decreased cardiorespiratory reserves.[10–12]
